# Le pied de Madura

**DOI:** 10.11604/pamj.2013.14.24.2381

**Published:** 2013-01-16

**Authors:** Fadwa El Amrani

**Affiliations:** 1 Service de Dermatologie, CHU Ibn Sina, Université Med V, Souissi, Rabat, Maroc

**Keywords:** Pied de Madura, Madurella mycetomi, mycétome, Madura foot, Madurella mycetomi, mycetoma

## Image in medicine

Le pied de Madura ou mycétome est une infection chronique dûe à des agents pathogènes fongiques ou bactériens, qui produisent des grains. Il se voit surtout dans les pays tropicaux et subtropicaux. Les mycétomes sont connus par leur localisation podale élective. L’évolution lente et progressive des lésions des téguments et des parties molles finit souvent par une atteinte secondaire du squelette sous-jacent. On distingue deux groupes: les eumycétomes (d'origine fongique) et les actinomycétomes (dûs à des bactéries aérobies). La distinction entre les deux est importante, puisque leurs traitements sont différents. Des combinaisons d'antibiotiques sont indiquées dans les actinomycétomes tandis que les imidazolés sont utilisés dans le traitement des eumycétomes. La chirurgie reste indiquée lorsque le traitement médical des eumycétomes est inefficace. Nous rapportons le cas d'un patient marocain de 50 ans, sans antécédent pathologique notable, n'ayant particulièrement pas séjourné en zone tropicale, qui consulte pour une tuméfaction indolore du pied, apparue dans les suites d'une blessure du dos du pied, ayant progressivement augmenté de taille depuis 20 ans. L'examen clinique retrouve une tuméfaction du dos du pied gauche, dure et polyfistulisée. L'examen microbiologique du liquide d'aspiration des trajets fistuleux et l'examen anatomopathologique d'une biopsie cutanée ont mis en évidence des grains noirs témoignant de leur origine fongique avec identification de Madurella mycetomi. La radiographie standard et l'IRM du pied n'ont pas montré de lyse osseuse. Un traitement à base de kétoconazole 200 mg/j a été entrepris, avec une légère diminution de la tuméfaction après 4 mois de traitement.

**Figure 1:**
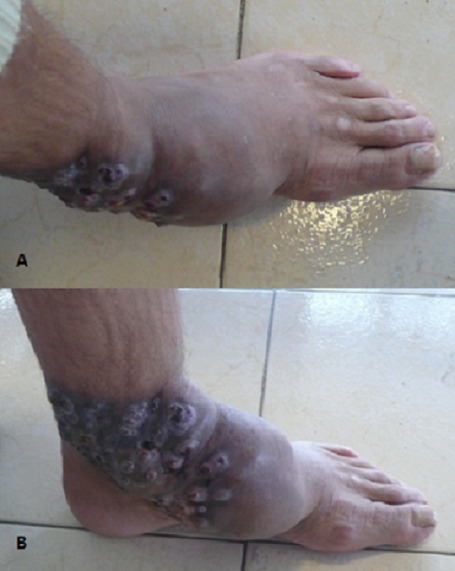
Tuméfaction dorso-latérale du pied, bien limitée, avec un placard polyfistulisé. (A) vue supérieure (B) vue latérale

